# Selective inhibition of sterol*O*-acyltransferase 1 isozyme by beauveriolide III in intact cells

**DOI:** 10.1038/s41598-017-04177-8

**Published:** 2017-06-23

**Authors:** Taichi Ohshiro, Keisuke Kobayashi, Mio Ohba, Daisuke Matsuda, Lawrence L. Rudel, Takashi Takahashi, Takayuki Doi, Hiroshi Tomoda

**Affiliations:** 1 Graduate School of Pharmaceutical Sciences, Kitasato University, Tokyo, 108-8641 Japan; 2 Section on Lipid Sciences, Department of Internal Medicine, Wake Forest School of Medicine, Winston-Salem, NC 27157 USA; 3 Faculty of Pharmaceutical Sciences, Yokohama College of Pharmacy, Kanagawa, 245-0062 Japan; 4 Graduate School of Pharmaceutical Sciences, Tohoku University, Sendai, 980-8578 Japan

## Abstract

Beauveriolide III (BeauIII) inhibited sterol *O*-acyltransferases 1 and 2 (SOAT1 and SOAT2), which are endoplasmic reticulum (ER) membrane proteins, in an enzyme-based assay, and selectively inhibited SOAT1 in a cell-based assay using SOAT1-/SOAT2-CHO cells. This discrepancy in SOAT inhibition by BeauIII was investigated. In the enzyme-based assay, BeauIII inhibited SOAT1 and SOAT2 to a similar extent using microsomes prepared from cells disrupted under the strongest sonication condition. In semi-intact SOAT1-/SOAT2-CHO cells prepared by a treatment with digitonin (plasma membrane permeabilized), BeauIII selectively inhibited SOAT1 (IC_50_; 5.0 µM (SOAT1) vs >90 µM (SOAT2)), while in those treated with saponin (plasma membrane and ER membrane permeabilized), BeauIII inhibited SOAT1 (IC_50_, 1.8 µM) and SOAT2 (5.9 µM). SOAT1-selective inhibition by BeauIII was reproduced in intact ER fractions prepared from SOAT1/SOAT2-CHO cells. A Western blotting analysis revealed that biotin-labeled beauveriolide bound to the SOAT1 protein prepared from SOAT1-CHO cells. We concluded that BeauIII binds to a putative active site responsible for SOAT1 that is located on the cytosolic side of the ER, while BeauIII is not accessible to the corresponding active site for SOAT2 located on the luminal side.

## Introduction

**Figure 1:**
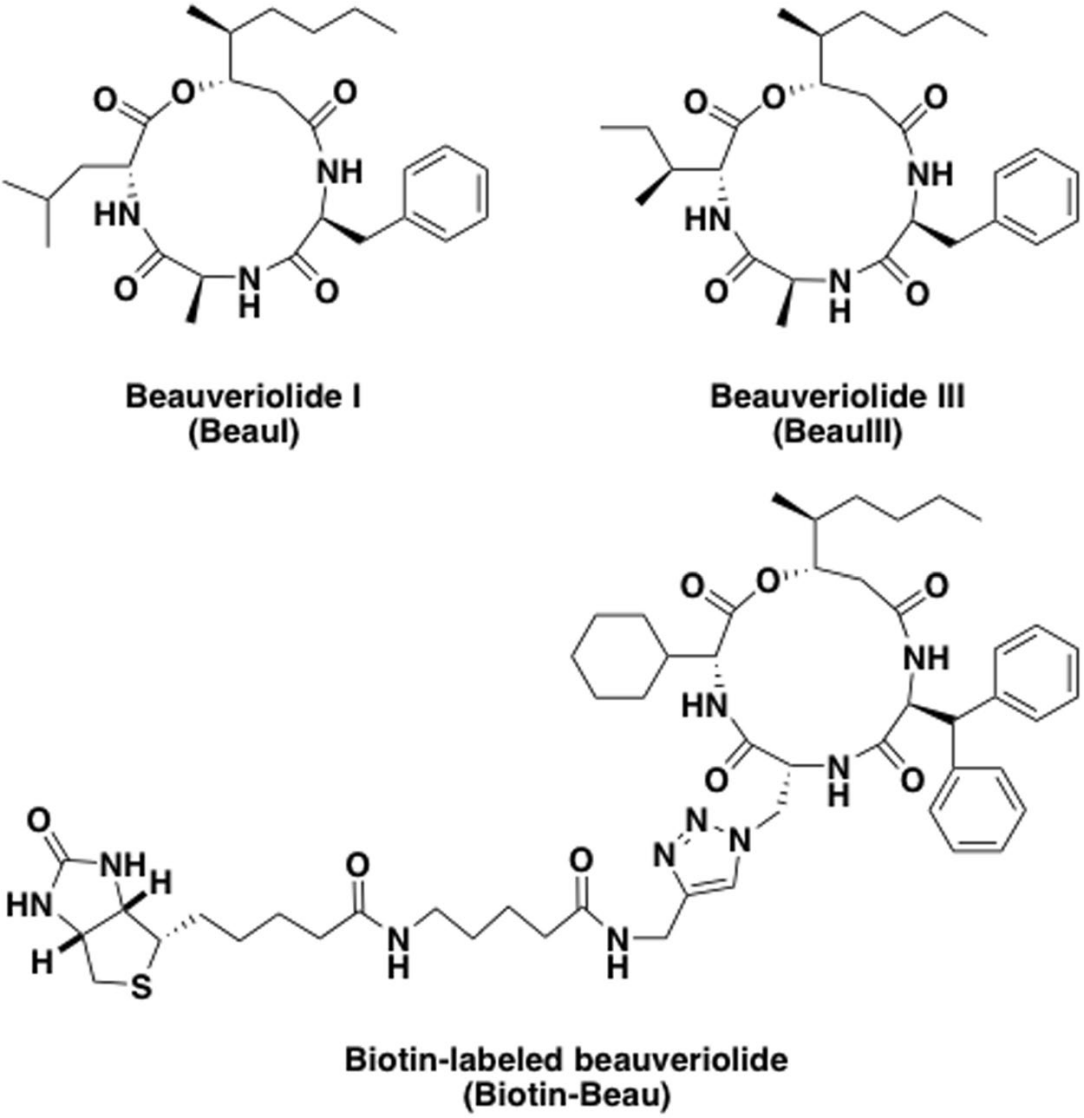
Structures of beauveriolides and biotin-labeled beauveriolide.

Beauveriolides, fungal metabolites produced by *Beauveria* sp. FO-6979, were identified as inhibitors of lipid droplet formation in mouse peritoneal macrophages^1–3^. A study of their mechanism of action revealed that beauveriolides inhibit the synthesis of cholesteryl ester (CE), one of the main constituents of lipid droplets in macrophages, by blocking sterol *O*-acyltransferase (SOAT, also known as acyl-CoA:cholesterol acyltransferase (ACAT)) activity^4^. Among eight beauveriolides^5^, beauveriolides I and III (BeauI and BeauIII, Fig. 1) were found to be the most potent SOAT inhibitors. More importantly, BeauIII was demonstrated to be orally active in atherosclerogenic mouse models, reducing atherosclerotic lesions in the aortae and hearts of apolipoprotein E knockout mice and in low density lipoprotein receptor knockout mice^1, 4^. Furthermore, we prepared a number of beauveriolide derivatives using combinatorial chemistry^1^ to examine structure-activity relationships and identified more potent inhibitors^1, 6–12^. During the derivative study, biotin-labeled beauveriolide (Biotin-Beau, Fig. 1) was synthesized and proven to be a useful probe for investigating the functions of SOAT^11^.

The enzyme SOAT (EC 2.3.1.26), which catalyzes the synthesis of CE from free cholesterol and long-chain fatty acyl-CoA, is regarded as a promising target for the treatment or prevention of atherosclerosis and hypercholesterolemia^6, 13, 14^. Two SOAT isozymes, SOAT1 and SOAT2, were recently identified in various mammals. SOAT1 is ubiquitously expressed in tissues and cells, while SOAT2 is predominantly expressed in the liver (hepatocytes) and intestines^13^. Both SOAT isozymes are membrane proteins localized in the endoplasmic reticulum (ER), and are assumed to possess several hydrophobic domains that penetrate the ER membrane^15, 16^. However, a consensus does not yet exist for a structural model of SOAT1 and SOAT2 proteins.

Rudel and co-workers established sophisticated cell lines including Chinese hamster ovary (CHO) cells expressing the SOAT1 or SOAT2 gene from the African Green monkey. (SOAT1-CHO cells and SOAT2-CHO cells, respectively) or humans (hSOAT1-CHO cells and hSOAT2-CHO cells, respectively)^17^. We previously examined the selectivity of microbial SOAT inhibitors toward SOAT1 and SOAT2 in enzyme- and cell-based assays using SOAT1-/SOAT2-CHO cells^18^. BeauI and BeauIII inhibited SOAT1 and SOAT2 to a similar extent in the enzyme-based assays using microsomes as an enzyme source, and selectively inhibited SOAT1 in the intact cell-based assay^18^. However, the reason for this discrepancy in SOAT inhibition by BeauI and BeauIII between the enzyme- and intact cell-based assays is unclear.

In the present study, we investigated SOAT isozyme inhibition by BeauIII in semi-intact (perforated) CHO cells and an intact ER fraction prepared from CHO cells in an attempt to explain this discrepancy.

## Results

### Selectivity of beauveriolides toward SOAT isozymes in cell- and enzyme-based assays

We previously reported that BeauI and BeauIII selectively inhibited SOAT1 in an intact cell-based assay, and inhibited SOAT1 and SOAT2 in a microsome assay as an enzyme source using SOAT1-/SOAT2-CHO cells. When hSOAT1-/hSOAT2-CHO cells were used, the same results were reproduced^18^; SOAT1-selective inhibition by BeauI and BeauIII in an intact cell-based assay with selectivity indexes (SI; log[IC_50_ for SOAT1]/[IC_50_ for SOAT2]) of <−1.52 and <−1.59 (SOAT1-selective inhibition; SI <−1.00), and SOAT1 and SOAT2 inhibition in an enzyme assay with SI of 0.46 and 0.13 (dual inhibition; −1.00 ≤ SI ≤+1.00), respectively (Table 1).

**Table 1:** Selectivities of beauveriolides toward SOAT isozymes.

Inhibitor	Assay system^a^	Species^b^	IC_50_ (µM)		SI (type)^c,d^	Reference^e^
			SOAT1	SOAT2		
Beauveriolide I (BeauI)	C	H	0.61	>20	<−1.52 (SOAT1)	this study
	E	H	3.2	1.1	0.46 (dual)	this study
	C	AGM	0.60	20	−1.52 (SOAT1)	18
	E	AGM	2.2	1.9	0.06 (dual)	18
Beauveriolide III (BeauIII)	C	H	0.51	>20	<−1.59 (SOAT1)	this study
	E	H	1.2	0.89	0.13 (dual)	this study
	C	AGM	0.90	>20	<−1.35 (SOAT1)	18
	E	AGM	3.0	3.0	0.00 (dual)	18
Biotin-labeled beauveriolide (Biotin-Beau)	C	AGM	>10	>10	NC^f^	11
	E	AGM	0.60	8.0	−1.12 (SOAT1)	11

^a^C: cell-based assay; E: enzyme-based assay.

^b^H: Human; AGM: African green monkey.

^c^Selectivity index (SI): log [IC_50_ for SOAT1/IC_50_ for SOAT2].

^d^Based on SI values, SOAT inhibitors were classified into three groups: dual type, −1.00 ≤ SI ≤ + 1.00; SOAT1-selective type, SI < −1.00; SOAT2-selective type, +1.00 < SI.

^e^IC_50_s values are cited from the reference.

^f^NC: not calculated.

**Figure 2:**
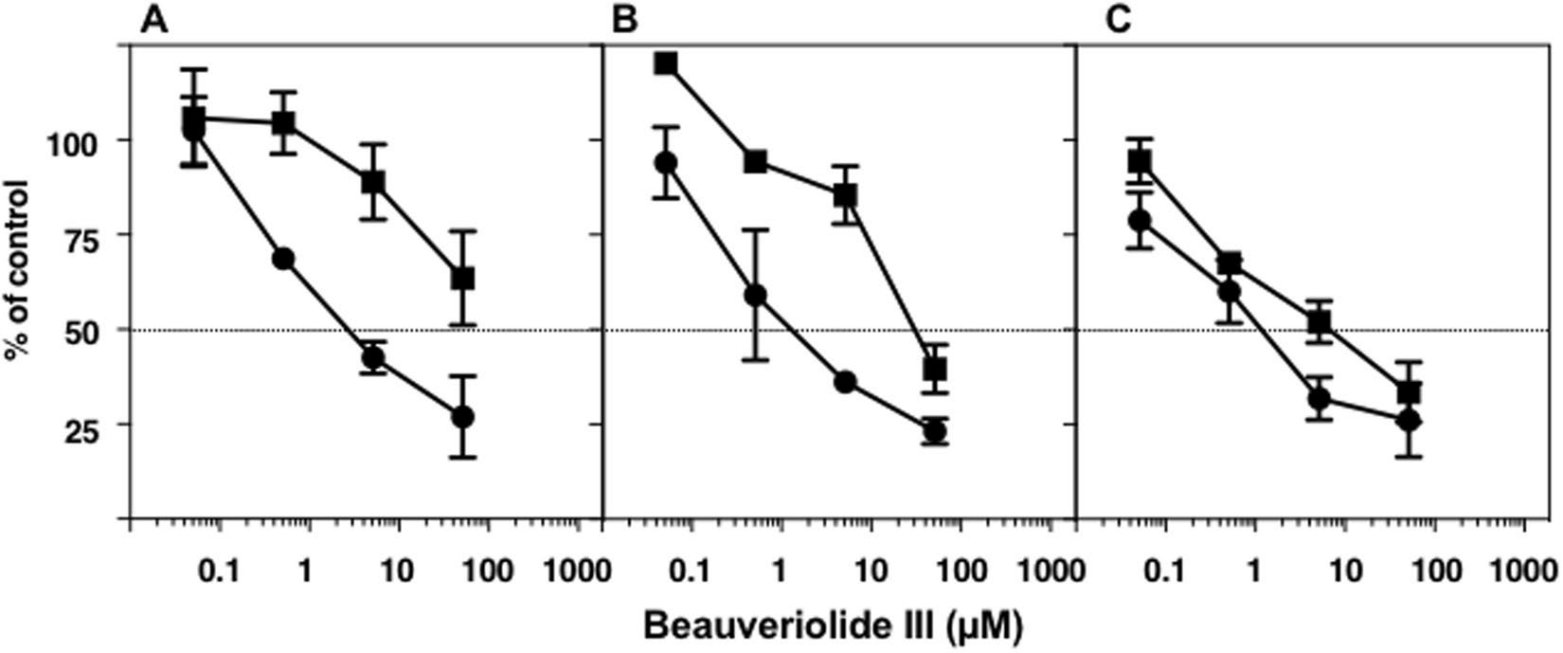
Inhibition of SOAT1 and SOAT2 by BeauIII in an enzyme-based assay using microsomes from SOAT1- and SOAT2-CHO cells disrupted by sonication. SOAT1-/SOAT-CHO cells were sonicated for 3 (**A**), 5 (**B**), and 10 min (**C**). Microsomes were prepared from the disrupted cells, and used as enzyme sources for the SOAT1 (●) and SOAT2 (■) assays. The results obtained were plotted as % of control (without drugs). Values represent means ± SD (n = 3~4).

In the enzyme assays, the cell lines were disrupted with a Potter-type homogenizer, and microsomes were prepared from the disrupted cells for use as an enzyme source for the SOAT assay. Our hypothesis is that ER membrane integrity is responsible for the SOAT inhibition selectivity of beauveriolides; in other words, the localization of SOAT active sites in ER membranes, which associate with inhibitors, is distinct in SOAT1 and SOAT2. In order to demonstrate our hypothesis, we investigated the effects of CHO-cell-disrupting conditions on BeauIII selectivity in an enzyme assay. SOAT1- and SOAT2-CHO cells were sonicated for 3 (a total of 3 min, 30-sec sonication and a 30-sec interval), 5, and 10 min, and microsomes were then prepared from cells for use as an enzyme source for the assay. As shown in Fig. 2, SOAT1 activity was inhibited by BeauIII with similar IC_50_s (2.7, 1.3, and 1.2 µM, respectively), while SOAT2 activity was disruption time-dependently inhibited by BeauIII with IC_50_s of >51, 31, and 6.6 µM, respectively. These results suggest that the strongest condition for cell disruption perturbed the ER membrane integrity (the ER membrane may turn inside out and outside in), thereby allowing BeauIII to access the active site of the SOAT2 isozyme in the damaged ER membrane.

### Selectivity of BeauIII toward SOAT isozymes in semi-intact cells

**Figure 3:**
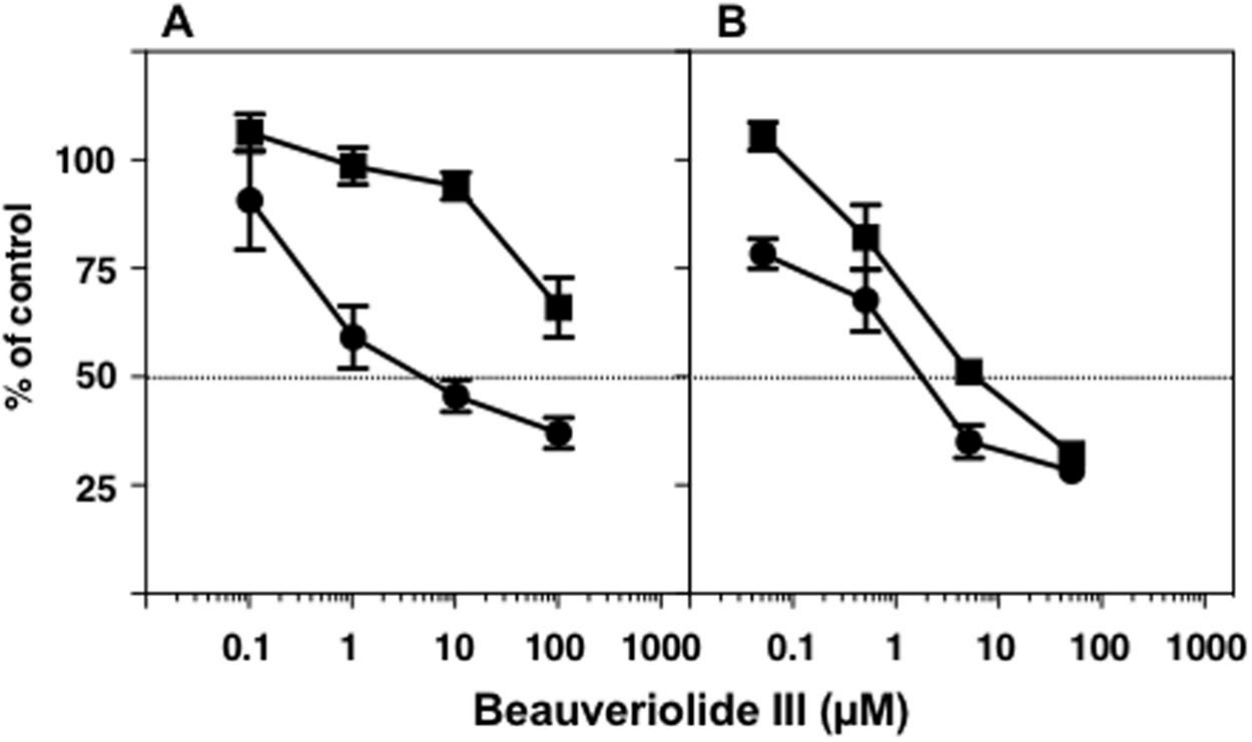
Inhibition of SOAT1 and SOAT2 by BeauIII in semi-intact SOAT1- and SOAT2-CHO cells. SOAT1-/SOAT2-CHO cells were permeabilized by digitonin (**A**) or saponin (**B**). [^14^C]CE was synthesized from [^14^C]oleoyl-CoA in semi-intact SOAT1- (●) and SOAT2-CHO (■) cells. The results obtained were plotted as % of control (without drugs). Values represent the means ± SD (n = 3~4).

In order to further demonstrate our hypothesis, BeauIII was tested in semi-intact (perforated) SOAT1- and SOAT2-CHO cells prepared by treatments with digitonin and saponin. The treatment of cells with digitonin only perforates the plasma membrane, which allows large molecules such as fatty acyl-CoA to permeabilize the plasma membrane^19^, whereas the treatment with saponin perforates the plasma membrane and ER membrane^20^. In digitonin-treated cells, the ER membrane structure was intact, allowing ER proteins such as SOAT to maintain their conformation, active site localization, and function normally^19^. In SOAT assays using semi-intact cells, [^14^C]CE was synthesized from the substrate [^14^C]oleoyl-CoA, but not from [^14^C]oleic acid because the substrates of acyl-CoA synthetase such as CoA and ATP are washed out of cells. As shown in Fig. 3, in digitonin-treated cells, BeauIII inhibited the SOAT1 isozyme with an IC_50_ of 5.0 µM, while the IC_50_ for SOAT2 was >90 µM, indicating that the inhibitor is selective to SOAT1 (SI, <−1.26). On the other hand, BeauIII inhibited SOAT1 and SOAT2 in saponin-treated cells, (SI, −0.52) as well as in the enzyme assay (SI, 0.00). Thus, the selective inhibition of BeauIII toward the SOAT1 isozyme was reproduced in digitonin-treated semi-intact cell-based assays, but was lost in saponin-treated semi-intact cells.

### Selectivity of BeauIII toward SOAT isozymes in intact ER fractions

**Figure 4:**
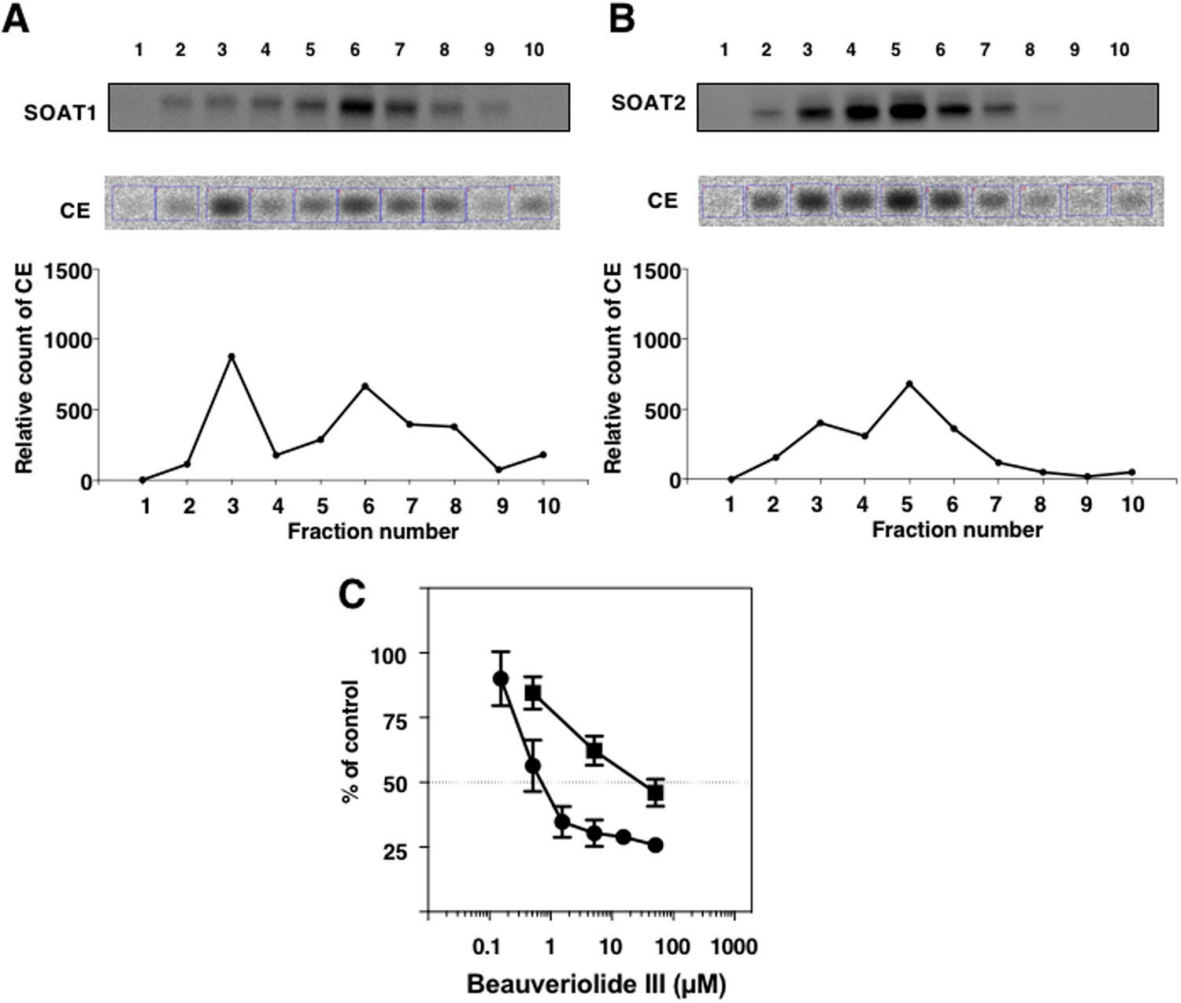
Inhibition of SOAT1 and SOAT2 by BeauIII in an enzyme-based assay using an intact ER fraction from SOAT1- and SOAT2-CHO cells sonicated for 3 min. Each cell lysate in SOAT1-/SOAT2-CHO cells sonicated for 3 min was fractionated in 10 fractions with the Optiprep^TM^ density gradient. In order to identify the ER fraction including SOAT proteins, SOAT protein levels and activities were measured using a Western blotting analysis and SOAT assay, respectively (**A** and **B**). The SOAT-rich ER fraction was then used as an enzyme source for the SOAT1 (●) and SOAT2 (■) assays (**C**). The results obtained were plotted as % of control (without drugs). Values represent means ± SD (n = 3~4).

In order to further demonstrate our hypothesis, we attempted to purify intact ER fractions from SOAT1- and SOAT2-CHO cells. SOAT1-/SOAT2-CHO cells were disrupted by a 3-min sonication, and cell lysates were fractionated in 10 fractions (fr. 1 to fr. 10) with an Optiprep^TM^ density gradient^21^. The measurement of SOAT activity and a Western blot analysis of SOAT proteins in each fraction were performed to identify the ER fraction containing SOAT proteins. As shown in Fig. 4A and B, fractionation patterns were very similar between SOAT1- and SOAT2-CHO cells. SOAT activity correlated well with SOAT proteins. In the SOAT assay using intact ER fractions, fr. 6 (SOAT1-CHO cells) and fr. 5 (SOAT2-CHO cells) were used as an enzyme source to investigate the inhibition of BeauIII. As shown in Fig. 4C, BeauIII inhibited the SOAT1 isozyme with an IC_50_ of 0.71 µM, and the SOAT2 isozyme with an IC_50_ of 29 µM, confirming SOAT1-selective inhibition by BeauIII (SI, −1.61) in intact ER fractions.

### Interaction between beauveriolide and the SOAT1 protein

**Figure 5:**
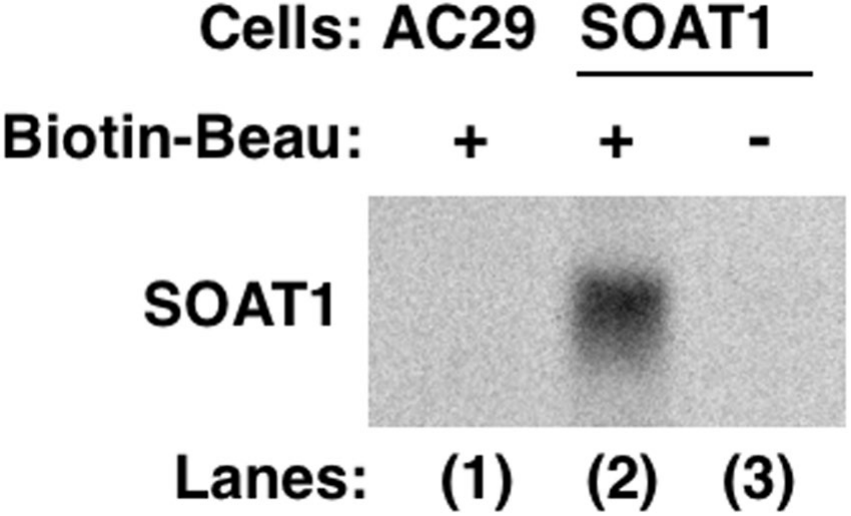
Analysis of the interaction between the SOAT1 protein and Biotin-Beau. Biotin-Beau-immobilized beads were used to analyze the interaction between beauveriolide and the SOAT1 protein. Bound proteins were analyzed using SDS-PAGE and Western blotting.

The direct interaction between beauveriolide and SOAT1 was investigated. Biotin-Beau^11^ showed no effect on CE synthesis in the cell-based assay using SOAT1-/SOAT2-CHO cells (Table 1) because of its low permeability in the plasma membrane. However, Biotin-Beau selectively inhibited SOAT1 in the enzyme assay (SI, −1.12) (Table 1). Therefore, Biotin-Beau may be employed as a useful bio-probe to investigate interactions between SOAT1 and beauveriolide. Biotin-Beau was attached to acrylamide-based streptavidin beads, and microsomes prepared from SOAT1-CHO cells and AC29 cells (control, SOAT-deficient CHO cells) were applied to the beads. After washing the beads, bound proteins were recovered with Laemmli sample buffer, separated by SDS-PAGE, and stained with the SOAT1 antibody^22^. As shown in Fig. 5, one band at approximately 53 kDa was clearly observed from SOAT1-CHO cells (lane (2)). However, no protein was observed from control AC29 cells (lane (1)) or control beads (no Biotin-Beau-carrying beads) (lane (3)). These results indicate that beauveriolide directly binds to the SOAT1 protein.

## Discussion

Our research group discovered a number of microbial SOAT inhibitors in several screening programs of an enzyme-based assay using rat liver microsomes and a cell-based assay using mouse peritoneal macrophages and SOAT1-/SOAT2-CHO cells^1, 23^. In most SOAT inhibitors tested, except for BeauI and BeauIII, IC_50_ values obtained in the cell-based assay and enzyme-based assay were consistent^18^. Inhibitors were classified into three groups from SI values; dual inhibitors (−1.00 ≤ SI ≤+1.00) inhibiting SOAT1 and SOAT2 (e.g. purpactins^18^, avasimibe^24^, and pactimibe^25^), SOAT1-selective inhibitors (SI, <−1.00) (e.g. Wu-V-23^17^ and K-604^26^), and SOAT2-selective inhibitors (SI, >+1.00) (e.g. pyripyropenes^27^). Interestingly, BeauI and BeauIII showed unusual characteristics in selectivity; dual inhibition in the enzyme-based assay versus SOAT1-selective inhibition in the intact cell-based assay (Table 1). This discrepancy prompted us to investigate the effects of beauveriolides on SOAT in more detail.

Intact cells generally maintain membrane structures and functions in every organelle in order to preserve cell integrity. However, when cells are disrupted by homogenization or sonication, membrane structures are altered to cause damage to intrinsic membrane integrity. We hypothesized that the discrepancy in the selectivity of inhibition of beauveriolides toward SOAT isozymes between the intact cell-based assay and enzyme-based assay may be due to altered membrane structures including the localization of the active sites of SOAT isozymes in the ER. The present study supported our hypothesis, namely, BeauIII accesses the active site of SOAT1, but cannot access the active site of SOAT2 in intact ER membranes, resulting in SOAT1-selective inhibition by BeauIII in the intact cell-based assay. These results imply that the methods of cell disruption markedly affect membrane structures and also that SOAT1 and SOAT2 have a distinct topology of active sites associated with BeauIII inhibition in ER membranes. We initially tested several conditions to disrupt SOAT1-/SOAT2-CHO cells. Among the conditions examined, our results strongly suggest that cell disruption conditions affect organelle integrity (Figs 2 and 3). Radhakrishnan *et al*. reported the importance of cell disruption conditions to obtain intact ER membranes^28^. We then attempted to obtain intact ER fractions from SOAT1-/SOAT2-CHO cells in order to confirm SOAT1-selective inhibition by BeauIII. When SOAT-rich ER fractions prepared from 3-min sonicated cells were used as an enzyme source, SOAT1-selective inhibition by BeauIII was reproduced (SI, −1.61) (Fig. 4C). We considered the cell fractionation procedure to have yielded intact ER fractions, although the SOAT fractions showed broad patterns (Fig. 4A and B). This may be because the ER is a heterogenous organelle; 3-min sonicated cells giving intact ER fractions with intrinsic functions to show SOAT1-selective inhibition by BeauIII. On the other hand, 10-min sonicated cells yielded sharp ER fractions at fr. 8 (SOAT1-CHO cells) and fr. 7 (SOAT2-CHO cells) by cell fractionation with the Optiprep^TM^ density gradient (Supplementary Fig. 1A and B), which are consistent with the reported patterns^28, 29^. However, SOAT1-selective inhibition by BeauIII became worse (SI, −1.04)(Supplementary Fig. 1C). These results strongly suggest that the ER is a labile and heterogenous organelle and also that usual cell disruption methods damage ER fractions. In the present study, we concluded that beauveriolide only penetrated the plasma membrane and accessed the active site of SOAT1, which faces the cytosolic side, but was unable to access the active site of SOAT2, which faces the luminal side of the ER membrane. BeauIII penetrated the plasma membrane, but not the ER membrane. ER membranes consist of phosphatidylcholine (>50%), cholesterol (<10%), and sphingomyelin (<10%), while plasma membranes have an almost equal quantity of the three lipids (20~30%)^30, 31^. This subtle compositional difference may affect the permeability of BeauIII. Otherwise, a specific transporter present in plasma membranes only may work on BeauIII.

The biological and structural properties of SOAT remain poorly understood because it is an ER membrane protein and very difficult to purify. Furthermore, the crystal structure of SOAT has not yet been reported. Using a molecular biological approach, several groups reported that SOAT proteins were predicted to possess several hydrophobic transmembrane domains^15, 16, 32–36^. Lin *et al*. showed that human SOAT1 had seven transmembrane domains in the ER, while human SOAT2 only had two^15, 32^. Furthermore, they suggested that the highly conserved FYXDWWN motif (403–409 in human SOAT1) was important for SOAT1 activity^33^. On the other hand, Joyce *et al*. demonstrated that African Green monkey SOAT1 and SOAT2 isozymes both had five transmembrane domains^16^. Das *et al*. recently showed that not only the FYXDWWN motif (377–383 in human SOAT2), but also the HEY motif (460–462 in human SOAT1 and 434–436 in human SOAT2) were essential for the activities of the SOAT1 and SOAT2 isozymes^36^. In addition, they found that some putative active site residues (serine, aspartic acid, histidine, and tyrosine) were essential for the activities of SOAT1 and/or SOAT2 using a point mutation technique^36^. Although there does not appear to be a consensus on the structural model for SOAT isozymes, these results indicate that active site topology in the ER membrane is distinct between SOAT1 and SOAT2.

In the present study, we demonstrated that Biotin-Beau is a practically useful bio-probe to investigate the interaction between SOAT1 and beauveriolide. In the analysis of beauveriolide-binding proteins using Biotin-Beau in microsomes prepared from SOAT1-CHO cells, the SOAT1 protein (53 KDa) was not visualized by Coomassie brilliant blue (CBB) staining. The reason for this may be that the expression level of the SOAT1 protein is very low in SOAT1-CHO cells. No significant differences were observed in CBB-stained proteins between AC29 cells (SOAT-deficient CHO cells) and SOAT1-CHO cells. Therefore, the application of the SOAT1 antibody allowed for the detection of SOAT1 as a beauveriolide-binding protein in the immunostaining analysis (Fig. 5). This is the first study to reveal the interaction of SOAT with an inhibitor. Furthermore, the cellular localization of beauveriolide and SOAT1 was investigated. Biotin-Beau was colocalized with a restricted area of SOAT1 protein distribution in mouse macrophages (Supplementary Fig. 2).

In summary, we herein elucidated the mechanisms underlying SOAT1-selective inhibition by BeauIII in intact cells and the interaction between SOAT1 and beauveriolide. Thus, microbial SOAT inhibitors are useful not only as a potential drug lead, but also as a bio-probe that will shed light on the unknown structures of SOAT isozymes.

## Materials and Methods

### Materials

BeauI and BeauIII were purified from the culture broth of the producing fungus *Beauveria* sp. FO-6979 according to our established methods (Supplementary Fig. 3)^1^. Biotin-labeled beauveriolide (Biotin-Beau) was synthesized as reported previously (Supplementary Fig. 3)^11^. [1-^14^C]Oleic acid (2.19 GBq/mmol) and [1-^14^C]oleoyl-CoA (1.85 GBq/mmol) were purchased from PerkinElmer Life and Analytical Sciences (Waltham, MA). 2.5% Trypsin and acrylamide-based streptavidin beads (Streptavidin UltraLink Resin) were purchased from Thermo Fisher Scientific (Waltham, MA). Triton X-100 was purchased from Sigma-Aldrich (St. Louis, MO). Digitonin were purchased from Wako Pure Chemical Industries (Tokyo, Japan). Saponin was purchased from MP Biomedicals (Santa Ana, CA). OptiPrep^TM^ was purchased from Axis-Shield (Luna Place, Scotland). An anti-rabbit IgG conjugated to horseradish peroxidase (HRP) was purchased from Medical & Biological Laboratories (Nagoya, Japan).

### Culture of SOAT1- and SOAT2-CHO cells

CHO cells (AC29 cells, SOAT-deficient cells)^37^ expressing the SOAT1 or SOAT2 gene from African Green monkeys and humans were cultured by the method described previously^17^.

### Assay for [^14^C]CE synthesis in intact SOAT1-/ SOAT2-CHO cells

The assay for the synthesis of [^14^C]cholesteryl ester (CE) from [^14^C]oleic acid in intact SOAT1- or SOAT2-CHO cells was performed according to our established method^18^.

### Preparation of an enzyme source from SOAT1-/SOAT2-CHO cells using a Potter-type homogenizer

An enzyme source from SOAT1-/SOAT2-CHO cells using a Potter-type homogenizer was prepared using our established method^18^. Briefly, SOAT1 or SOAT2-CHO cells (2.0 × 10^8^ cells) were homogenized in 5 ml cold buffered sucrose solution (pH 7.2, 100 mM sucrose, 50 mM KCl, 40 mM KH_2_PO_4_, and 30 mM EDTA, hereafter referred to as Buffer A) including protease inhibitors (Complete mini (Roche)) in the Potter-type homogenizer. The microsomal fraction was pelleted by centrifugation at 100,000 ×  *g* at 4.0 °C for 1.0 h (TLA110, Beckman Coulter), resuspended in the same buffer at a concentration of 5.0 mg protein/ml, and stored at −80 °C until used.

### Preparation of an enzyme source from SOAT1-/SOAT2-CHO cells using a sonication machine

SOAT1 or SOAT2-CHO cells (2.0 × 10^8^ cells) were sonicated with a Bioruptor (Biosonics Inc.) in 1 ml cold Buffer A including protease inhibitors for 3.0 (30-sec sonication and a 30-sec interval, three rounds), 5.0 (five rounds), and 10 (ten rounds) min on ice. After cell sonication, the sample as an enzyme source was centrifuged at 8,000 ×  *g* for 15 min to remove intact cells and debris and stored at −80 °C until used.

### Purification of an intact ER fraction with an OptiPrep^TM^ density gradient

OptiPrep^TM^ density gradients were performed according to the manufacturer’s instructions with minor modifications. OptiPrep (60% iodixanol) was diluted to a final concentration of 45% iodixanol, 100 mM sucrose, 50 mM KCl, 40 mM KH_2_PO_4_, and 30 mM EDTA, as the gradient stock solution. Briefly, SOAT1- or SOAT2-CHO cells (>90% confluent) were washed twice with PBS and then incubated with PBS (500 µl) with 0.0625% trypsin/EDTA at 37 °C for 5.0 min in 5.0% CO_2_. All subsequent operations were performed at 4 °C. After centrifugation at 500 ×  *g* for 5.0 min, cell pellets were resuspended in 500 µl of Buffer A and sonicated for 3.0 (30-sec sonication and a 30-sec interval, three rounds) and 10 (ten rounds) min on ice. Disrupted SOAT1- or SOAT2-CHO cells were centrifuged at 3,000 ×  *g* for 10 min to remove intact cells and debris. The supernatant was diluted to a total volume of 1.0 ml using Buffer A. A discontinuous Optiprep^TM^ gradient was generated in a tube by overlaying the following Optiprep^TM^ solutions all in Buffer A: 1.2 ml each of 10, 12.5, 15, 17.5, 20, 22.5, 25, 27.5, and 30% iodixanol. The gradient was centrifuged using himac CP70G (HITACHI) with a swinging bucket rotor (RPS40T, HITACHI) at 100,000 ×  *g* for 16 h and allowed to slow down without the application of a break. Then, one band of membrane was clearly visible in a tube. After centrifugation, 1.2 ml of the fraction was collected from the top of the tube, giving a total of 10 fractions. All 10 fractions of SOAT protein levels and activities were analyzed by an immunoblot analysis (described below) and SOAT assay (described below) to identify intact ER fractions including SOAT proteins. After identifying ER fractions, they were stored at −80 °C until used.

### Immunoblotting analysis of SOAT1 and SOAT2

An immunoblotting analysis of SOAT1 and SOAT2 was performed by our established method^22^. Briefly, all 10 fractions (15 μL) isolated with the OptiPrep^TM^ density gradient as described above were added to 2x Laemmli sample buffer and 100 mM dithiothreitol (DTT), and then incubated at room temperature for 30 min. Furthermore, 400 mM iodoacetamide (IAA) was added. After a 30-min incubation at room temperature, 5.0 µl of 1.0 M Tris/HCl (pH 9.2) was added to the mixture, a part of which was applied to SDS-PAGE (10% gel) at a constant current of 20 mA for 1.5 h. Proteins were transferred to a polyvinylidene difluoride membrane (PVDF, Immobilon-P, Millipore, USA) for 1.0 h at 100 V by a Western blot apparatus (MODEL BE-350, BIO CRAFT, Japan). After transfer, the PVDF membrane was blocked with 5.0% skim milk in TBST buffer (20 mM Tris/HCl (pH 7.4), 100 mM NaCl, and 0.10% (v/v) Tween 20) at room temperature for 1.0 h. The PVDF membrane was washed with TBST buffer and then soaked in the primary antibody (anti-SOAT1 or anti-SOAT2 rabbit polyclonal antibody^22^, 1:1000 dilution) in TBST buffer at room temperature for 1.0 h. The primary antibody was then removed, and the PVDF membrane was washed and soaked in the secondary antibody (anti-rabbit IgG conjugated to HRP, 1:1000 dilution) for 1.0 h. After removal of the secondary antibody, the blot was visualized using ECL Western Blotting Detection Reagents (GE Healthcare, USA) and detected using LAS-4000 mini with a Science Lab 2005 software (Fujifilm Co., Japan).

### Assay for SOAT1 and SOAT2 activities in microsomes or ER fractions

SOAT1 and SOAT2 activities were measured using fractionation prepared as described above as an enzyme source^18^. Briefly, an assay mixture was made containing 500 µg BSA (fatty acid free) and [1-^14^C]oleoyl-CoA (20 µM, 3.7 kBq). A test sample (5.0 µl in methanol solution) and microsomes or the ER fractionation of SOAT1- and SOAT2-CHO cells in a total volume of 200 µl Buffer A was incubated at 37 °C. The SOAT reaction was started by adding [1-^14^C]oleoyl-CoA. After a 5-min incubation, the reaction was stopped by adding chloroform: methanol (2:1, 1.2 ml). The product [^14^C]CE was extracted using the method of Bligh and Dyer^38^. After the organic solvent was removed by evaporation, lipids were separated on a TLC plate and the radioactivity of [^14^C]CE was measured by our established method^18^.

### Preparation of semi-intact SOAT1-/SOAT2-CHO cells

Semi-intact SOAT1- or SOAT2-CHO cells were prepared using the method previously described^19, 20^ with minor modifications. SOAT1-CHO or SOAT2-CHO cells collected from a culture in a 100-mm dish (80–90% confluent) were washed with PBS, and then incubated with PBS (1.0 ml) containing 0.0625 mg/ml trypsin at 37 °C for 5.0 min in 5.0% CO_2_. After the trypsin treatment, cells were collected after centrifugation at 4.0 °C for 10 min. Cells were then washed with ice-cold KHM (110 mM potassium acetate, 20 mM Hepes (pH 7.2), and 2.0 mM magnesium acetate), resuspended in KHM containing 50 µg/ml digitonin or 2.0% saponin to make a concentration at 5.0 × 10^6^ cells/ml, and incubated on ice for 5.0 min. An equal volume of KHM was added to terminate permeabilization, and some cells were stained with trypan blue to confirm that they were permeabilized. Perforated cells were collected by centrifugation at 1,000 rpm at 4.0 °C for 10 min and resuspended in ice-cold KH (90 mM potassium acetate and 50 mM Hepes (pH 7.2)). After a 10-min incubation on ice, cells were collected by centrifugation at 1,000 rpm at 4.0 °C for 10 min and resuspended in KHM to make a concentration at 5.0 × 10^6^ (digitonin) or 5.0 × 10^7^ (saponin) cells/ml for SOAT1-CHO and 1.0 × 10^6^ (digitonin) or 1.0 × 10^7^ (saponin) cells/ml for SOAT2-CHO cells.

### Assay for [^14^C]CE synthesis in semi-intact SOAT1-/SOAT2-CHO cells

SOAT1 and SOAT2 activities were assessed using semi-intact cells (perforated cells) prepared as described above. An assay mixture was made containing 500 µg BSA (fatty acid free) and [1-^14^C]oleoyl-CoA (20 µM, 3.7 kBq). A test sample (5.0 µl in methanol solution) and semi-intact SOAT1- or SOAT2-CHO cells (5.0 or 1.0 × 10^5^ cells permeabilized with digitonin and 5.0 or 1.0 × 10^6^ cells permeabilized with saponin) in a total volume of 200 µl KHM buffer were incubated at 37 °C. The SOAT reaction was initiated by the addition of [1-^14^C]oleoyl-CoA. Note that [^14^C]oleic acid did not work as a substrate for CE synthesis in this assay. After a 5-min incubation, the reaction was stopped by adding chloroform: methanol (2:1, 1.2 ml). The product [^14^C]CE was extracted by the method of Bligh and Dyer^38^. After the organic solvent was removed by evaporation, lipids were separated on a TLC plate and the radioactivity of [^14^C]CE was measured by our established method^18^.

### Analysis of the interaction of Biotin-Beau with the SOAT1 protein

The microsomal fraction (500 µg protein in 100 µl buffer A) prepared from AC29 (SOAT-deficient CHO cells) or amgSOAT1-CHO cells using the Potter-type homogenizer as described above were mixed with 0.10% (v/v) Triton X-100 on ice for 10 min. Biotin-Beau (10 nmol)^11^ was attached to acrylamide-based streptavidin beads (5.0 nmol)^39^. Microsomes (500 µg) prepared from AC29 cells or SOAT1-CHO cells were added to Biotin-Beau-carrying beads and mixed well at 4.0 °C for 1.0 h with a rotator. The beads were collected by centrifugation at 1,000 rpm at 4.0 °C for 5.0 min and washed three times with PBS (300 µl). The beads were then incubated at room temperature for 30 min in Laemmli sample buffer (50 µl) containing DTT (5.5 µl, 100 mM) in order to recover bound proteins. IAA (25.2 µl, 400 mM) was added to the bead mixture. After a 30-min incubation at room temperature, 2.0 µl of 1.0 M Tris-HCl (pH 9.2) was added to the bead mixture, a part of which was applied to SDS-PAGE (10% gel) at a constant current of 20 mA for 1.5 h. After separation by SDS-PAGE, SOAT1 protein levels was measured as described above.

## Electronic supplementary material

Supplementary information
