# Supplementary material for: Selective inhibition of sterolO-acyltransferase 1 isozyme by beauveriolide III in intact cells
Source: Sci Rep. 2017 Jun 23;7:4163. doi: 10.1038/s41598-017-04177-8 (PMC5482845; doi:10.1038/s41598-017-04177-8)
Supplement: Supplementary file 1 — Supplementary information [file 41598_2017_4177_MOESM1_ESM.pdf]

Supplementary information

Selective inhibition of sterol *O*-acyltransferase 1 isozyme by beauveriolide III in intact cells

Taichi Ohshiro[1], Keisuke Kobayashi[1], Mio Ohba[1], Daisuke Matsuda[1], Lawrence L. Rudel[2], Takashi Takahashi[3], Takayuki Doi[4], and Hiroshi Tomoda[1,*]

[1]Graduate School of Pharmaceutical Sciences, Kitasato University, Tokyo 108-8641, Japan; [2]Section on Lipid Sciences, Department of Internal Medicine, Wake Forest School of Medicine, Winston-Salem, NC 27157, USA; [3]Faculty of Pharmaceutical Sciences, Yokohama College of Pharmacy, Kanagawa 245-0062, Japan; [4]Graduate School of Pharmaceutical Sciences, Tohoku University, Sendai 980-8578, Japan

[*] To whom correspondence should be addressed: Hiroshi Tomoda, Graduate School of Pharmaceutical Sciences, Kitasato University, 5-9-1 Shirokane, Minato-ku, Tokyo 108-8641, Japan, E-mail: tomodah@pharm.kitasato-u.ac.jp

Supplementary Figure S1

Inhibition of SOAT1 and SOAT2 by BeauIII in an enzyme-based assay using an intact ER fraction from SOAT1- and SOAT2-CHO cells sonicated for 10 min.

Supplementary Figure S2

Co-localization of beauveriolide and SOAT1 in mouse peritoneal macrophages.

Supplementary Figure S3

UFLC charts of BeauI, BeauIII and Biotin-Beau.

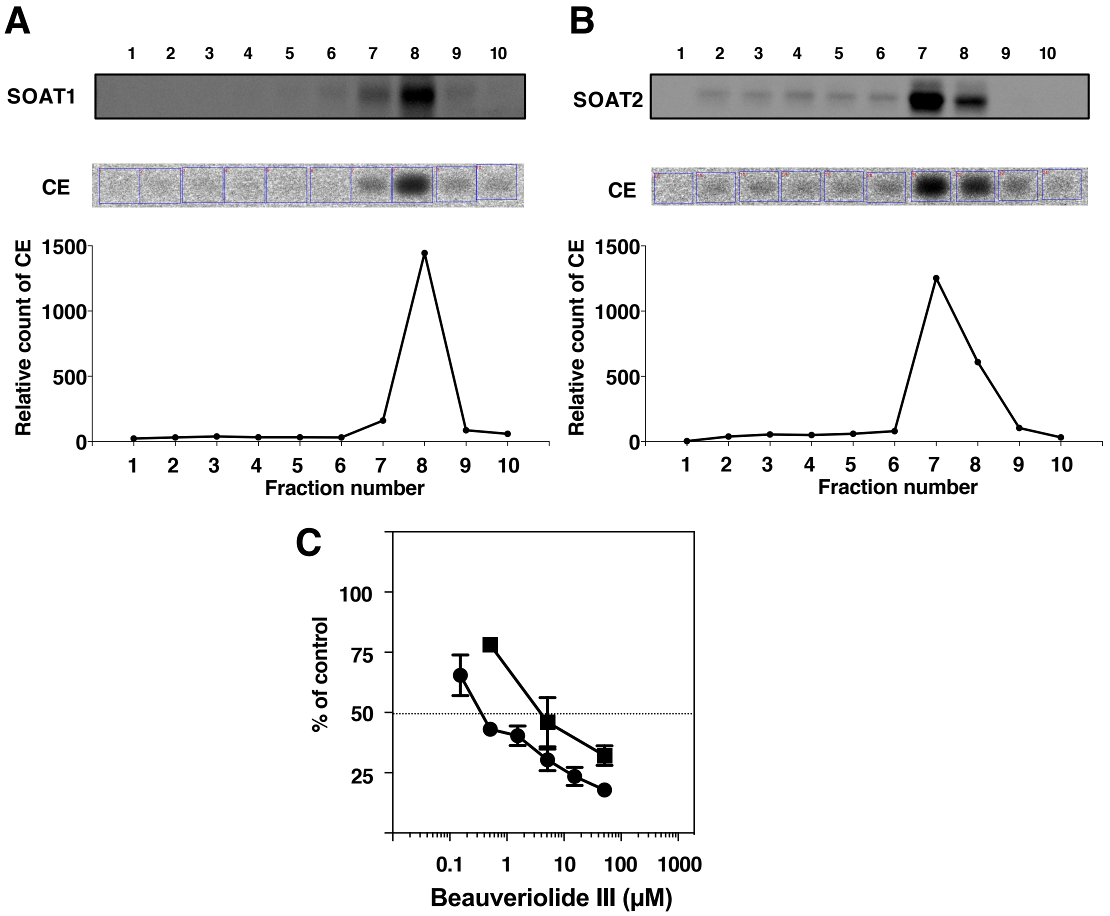

Supplementary Figure S1

Inhibition of SOAT1 and SOAT2 by BeauIII in an enzyme-based assay using an intact ER fraction from SOAT1- and SOAT2-CHO cells sonicated for 10 min.

Each cell lysate in SOAT1-/SOAT2-CHO cells sonicated for 10 min was fractionated in 10 fractions with the OptiprepTM density gradient. In order to identify the ER fraction including SOAT proteins, SOAT protein levels and SOAT activities were measured using a Western blotting analysis and SOAT assay, respectively (A and B). Then, SOATA-rich ER fraction was then used as an enzyme sources for SOAT1 (●) and SOAT2 (■) assas (C). The results obtained were plotted as % of control (without drugs). Values represent means ±SD (n=3~4).

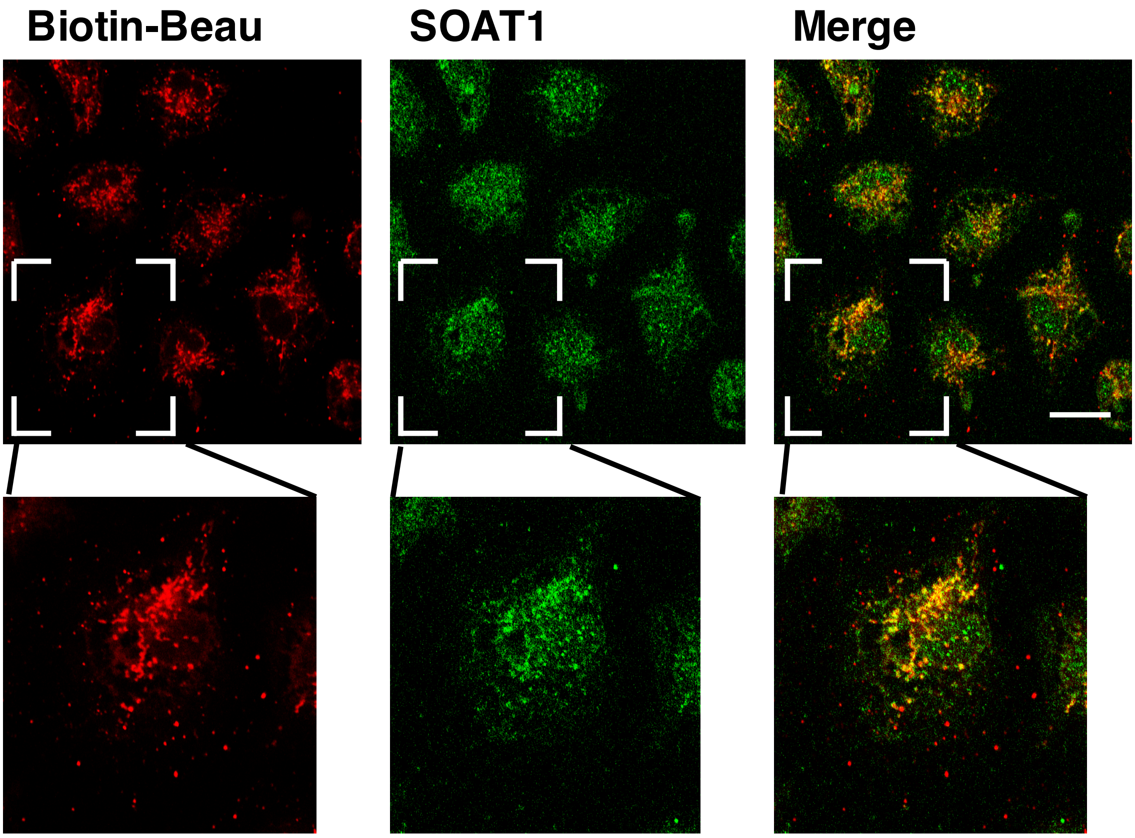

Supplementary Figure S2

Co-localization of beauveriolide and SOAT1 in mouse peritoneal macrophages.

Macrophages were double-stained with anti-Biotin-Beau (red) and anti-SOAT1 (green). Inserts show a magnification of the area indicated by the squares. Scale bars, 20 μm.

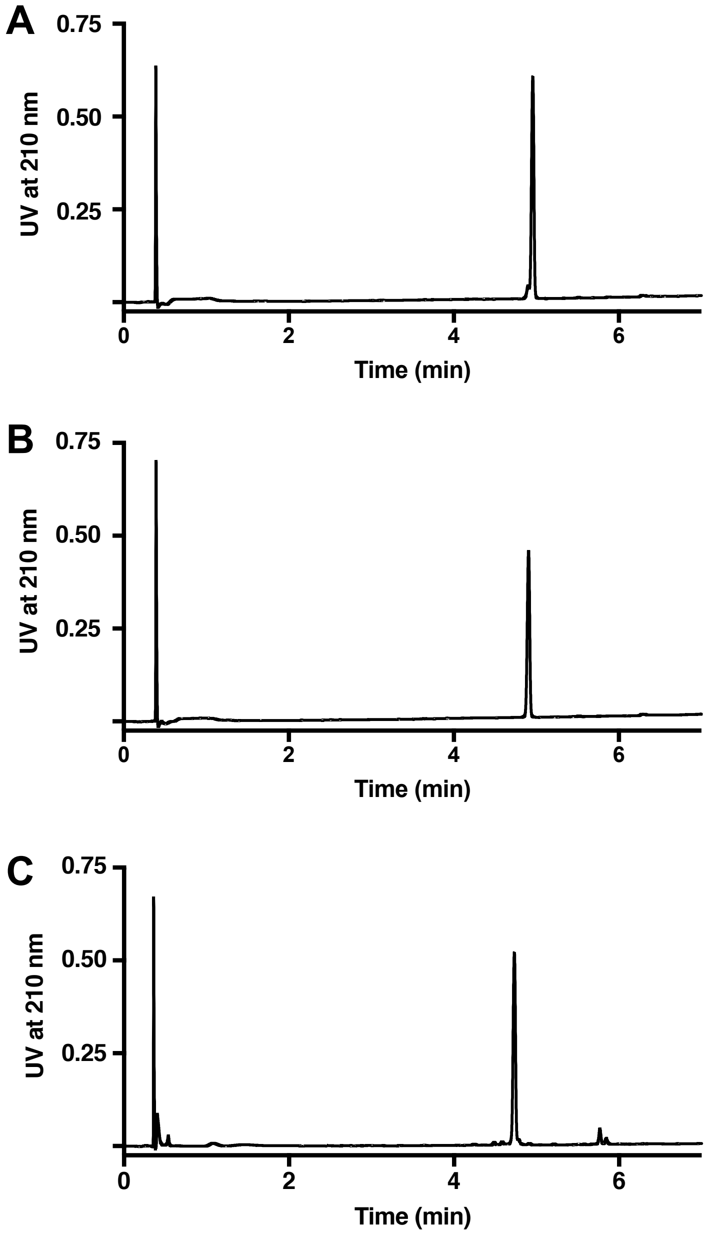

Supplementary Figure S3

UFLC charts of BeauI, BeauIII and Biotin-Beau.

BeauI (A), BeauIII and Biotin-Beau (C) were analyzed by ultra fast liquid chromatography (UFLC)

Supplementary Experiment Methods

Materials

Goat anti-rabbit IgG conjugated to fluorescein isothiocyanate (FITC) was purchased from Sigma-Aldrich (St. Louis, MO). Hank's balanced salt solution was purchased from Nissui Seiyaku (Tokyo, Japan). Streptavidin conjugated to Alexa Fluor 546 was purchased from Thermo Fisher Scientific (Waltham, MA). A tissue culture chamber (LAB-TEK 8-chamber) was purchased from Nulge Nunc (Rochester, NY).

Immunofluorescence staining of SOAT1 and Biotin-Beau in mouse peritoneal macrophages

Mouse peritoneal macrophages from ICR mice (Japan SLC, Inc., Japan) were prepared as described previously[1]. Briefly, primary mouse peritoneal macrophages (2.0 x 10^5 cells) with liposomes in each well of a tissue culture chamber (LAB-TEK 8-chamber) were incubated at 37°C in 5.0% CO_2. After a 14-h incubation, cells were washed three times with PBS, fixed with 3.7% formalin for 10 min, and permeabilized with PBS containing 0.10% Triton X-100 at room temperature for 20 min. After a brief rinse with PBS, cells were incubated with blocking buffer (3.0% BSA and 0.10% Triton X-100 in PBS) at room temperature for 1.0 h. Cells were then incubated with the anti-SOAT1 rabbit polyclonal antibody (1.5 μg/ml in blocking buffer)[2] and Biotin-Beau (9.8 μM)[3] at room temperature. After a 1-h incubation, cells were stained with the secondary antibody, a goat anti-rabbit IgG conjugated to FITC (10 μM) and streptavidin conjugated to Alexa Fluor 546 (1.0 μg/ml) in blocking buffer, for 1.0 h. Cells were washed three times after the incubation and analyzed with confocal laser scanning microscopy (LSM-510 META, Carl Zeiss, Germany). Images were analyzed with a Zeiss LSM Image Browser (Version 3.5.0.376)

UFLC analysis

BeauI, BeauIII and Biotin-Beau dissolved in methanol were analyzed by (UFLC) (Prominence, Shimadzu, Kyoto, Japan). under the following conditions: column, Shim Pack XR-ODS (Shimadzu), 2.0 x 75 mm; column temperature, 50°C; solvent, 6-min linear gradient from 5.0% acetonitrile in 0.10% phosphoric acid to 95% acetonitrile in 0.10% phosphoric acid; flow rate, 0.55 ml/min; detection, UV at 210 nm.

Supplementary References

1 Namatame, I., Tomoda, H., Arai, H., Inoue, K. & Omura, S. Complete inhibition of mouse macrophage-derived foam cell formation by triacsin C. *J Biochem* **125**, 319-327 (1999).

2 Lee, R. G., Willingham, M. C., Davis, M. A., Skinner, K. A. & Rudel, L. L. Differential expression of ACAT1 and ACAT2 among cells within liver, intestine, kidney, and adrenal of nonhuman primates. *J Lipid Res* **41**, 1991-2001 (2000).

3 Doi, T. *et al*. Conformationally restricted analog and biotin-labeled probe based on beauveriolide III. *Bioorg Med Chem Lett* **22**, 696-699 (2012).
